# 2,3,7,8-Tetrachlorodibenzo-p-dioxin-induced aryl hydrocarbon receptor activation enhanced the suppressive function of mesenchymal stem cells against splenocyte proliferation

**DOI:** 10.1007/s11626-019-00383-y

**Published:** 2019-08-05

**Authors:** Guorui Zhang, Xiaoming Li, Yi Cheng, Haiyang Yu, Wen Gu, Zhilei Cui, Xuejun Guo

**Affiliations:** Department of Respiratory Medicine, Xinhua Hospital Affiliated to Shanghai Jiao Tong University School of Medicine, Shanghai, China

**Keywords:** Aryl hydrocarbon receptor, Inducible nitric oxide synthase, Mesenchymal stem cell, Nitric oxide, Proliferation

## Abstract

The immunosuppressive function of mesenchymal stem cells (MSCs) is well known. Aryl hydrocarbon receptor (AhR), a transcription factor of the bHLH/PAS family, is widely expressed in several cells and is involved in various physiological and pathological processes. Previously, we found that the expression of AhR was downregulated in MSCs isolated from mice with neutrophilic asthma and that the activation of AhR enhanced the function of MSCs to alleviate neutrophilic asthma. We hypothesized that AhR activation enhanced MSCs for their immunosuppressive function. We aimed to investigate whether AhR activation can augment the suppressive function of MSCs against splenocyte proliferation. We co-cultured MSCs or AhR-activated MSCs with splenocytes at different ratios. The results showed that AhR activation in MSCs upregulated the expression of inducible nitric oxide (iNOS), which promoted the production of nitric oxide (NO), thus enhancing the inhibitory effect on splenocyte proliferation. The NO donor S-nitroso-N-acetylpenicillamine also inhibited the proliferation of splenocytes, and the iNOS inhibitor N(G)-nitro L-arginine methyl ester and NO scavenger 2-phenyl-4,4,5,5-tetramethylimidazoline-1-oxyl 3-oxide partially reversed the immunosuppressive function. Our study indicates that the AhR activation of MSCs might have an important role in the regulation of splenocyte proliferation and might serve as a potential strategy for treating immune-related diseases.

## Introduction

Bone marrow-derived mesenchymal stem cells (MSCs) are pluripotent stem cells that play an important role in regenerative therapy (Uder et al. 2018). MSCs modulate the immunological responses involved in several inflammatory and immune-related diseases. The molecular mechanism by which MSCs exert their immunosuppressive function has been illustrated. Nitric oxide (NO) (Sato et al. 2007; Jianjun et al. 2013; Kim et al. 2016), indoleamine 2,3-dioxygenase (IDO) (Meisel et al. 2004; Milosavljevic et al. 2018), transforming growth factor-β (TGF-β) (de Araujo Farias et al. 2018), interleukin (IL)-10 (Najar et al. 2016), prostaglandin E2 (PGE2) (Lin et al. 2012), and tumor necrosis factor-stimulated gene 6 (TSG-6) (Wang et al. 2018) have been reported to mediate the suppressive function of MSCs. Different results may be associated with the isolation and culture condition of MSCs and different circumstances of the diseases. While many studies have showed that the mechanism of immunosuppressive function of MSCs varied among different species (Ren et al. 2009; Su et al. 2014). Human-, pig-, and monkey-derived MSCs utilize IDO for immunosuppression, while rat-, rabbit-, hamster-, and mouse-derived MSCs utilize NO. Aryl hydrocarbon receptor (AhR), a ligand-dependent transcription factor of the bHLH/PAS family, mediates several important physiological processes in response to exogenous substances and natural compounds such as tryptophan metabolites, dietary components, and microbial community–derived factors (Quintana and Sherr 2013; Cella and Colonna 2015). 2,3,7,8-Tetrachlorodibenzo-p-dioxin (TCDD), the most potent agonist of AhR, has been investigated in depth (Schmidt and Bradfield 1996). Previously, we found that the expression of AhR was downregulated in MSCs isolated from mice with neutrophilic asthma and that the activation of AhR in MSCs alleviated the neutrophilic airway inflammation in vivo (data unpublished). We hypothesized that AhR activation could improve the immunosuppressive function of MSCs. In this study, we aimed to investigate whether AhR activation can improve the inhibitory function of MSCs against splenocyte proliferation.

## Materials and Methods

### **Mice**

Four-week-old female C57BL/6 mice were purchased from Shanghai SLAC Corporation (Shanghai, China). The mice were sacrificed using a lethal dose of sodium, with all efforts being made to minimize suffering as much as possible. All animal care and handling protocols were approved by the Ethics Committee of Xinhua Hospital Affiliated to Shanghai Jiao Tong University School of Medicine. All experiments were carried out in accordance with the Guide for the Care and Use of Laboratory Animals.

### **Isolation, culture, and identification of MSCs**

MSCs were isolated from the femurs and tibias of mice. Briefly, the femurs and tibias were flushed with pre-cold phosphate-buffered saline (PBS) until they turned white. Thereafter, the cell suspension was passed through a 70-μm cullender (Falcon, Corning, NY) to obtain MSCs. The obtained MSCs were cultured for 3 days in 100-mm culture dishes with low-glucose Dulbecco’s modified Eagle’s medium (Hyclone, Waltham, MA) containing 10% fetal bovine serum and 1% 100 U/mL penicillin and 100 μg/mL streptomycin (Gibco, Waltham, MA) in an incubator. The non-adherent cells were discarded, and MSCs were trypsinized to obtain plastic-adherent cells. MSCs in the fourth through tenth passages were used in the subsequent experiments. They were identified by fluorescence-activated cell sorting (FACS) using anti-CD29-FITC, anti-Sca-1-PE, anti-CD105-PE, anti-CD90-FITC, anti-CD45-FITC, anti-CD11b-PE, anti-CD34-PE, and anti-CD80-PE antibodies (eBioscience, Waltham, MA).

MSCs were treated with 1 nM TCDD (AccuStandard, New Haven, CT) for 24 h to activate AhR and harvested for the subsequent experiments. Both MSCs and AhR-activated MSCs were stimulated by TNF-α and IFN-γ (PeproTech, Rocky Hill, NJ) at a concentration of 2 μg/mL to induce immunosuppression in the co-culture experiments.

### **Mitomycin C–mediated suppression of MSC proliferation**

During the co-culture of MSCs and splenocytes, the proliferation of MSCs will consume the culture medium and then affecting splenocyte proliferation. It is hard to distinguish the immunosuppressive function of MSCs and the effect of their own proliferative activities. Therefore, we need to remove the proliferation of MSCs with the use of mitomycin C (MedChemExpress, Monmouth Junction, NJ ). The optimal concentration of mitomycin C was determined. Briefly, mitomycin C at different concentrations was added to the MSC culture medium and then, the cells were incubated in a water bath at 37°C for 30 min. Thereafter, MSCs were maintained in fresh complete medium for 0, 24, 48, and 72 h. The proliferation of MSCs was assessed using a cell counting kit-8 (Dojindo, Kumamoto, Japan), and the optical density (OD) was obtained using multiscan spectrum. The proliferation of MSCs and AhR-activated MSCs was suppressed using the optimal concentration of mitomycin C for the subsequent co-culture experiments.

### **Splenocyte isolation**

The mice were sacrificed by cervical dislocation. The spleen was removed aseptically and placed in pre-cold PBS. The spleen samples were ground using a syringe piston and passed through a 70-μm cullender. The cells were resuspended in PBS and isolated by density gradient centrifugation. Splenocytes were maintained in Roswell Park Memorial Institute 1640 medium (Hyclone) containing 10% fetal bovine serum and 1% 100 U/mL penicillin and 100 μg/mL streptomycin.

### **Carboxyfluorescein succinimidyl amino ester cell labeling**

Splenocytes were washed and resuspended to 5–10 × 10^6^/mL concentration in pre-warmed PBS. Carboxyfluorescein succinimidyl amino ester (CFSE) (eBioscience, MA) was added to a final concentration of 1 μM, mixed immediately, and incubated for 10 min at 25°C in the dark. The labeling of splenocytes was stopped by the addition of 4–5 volumes of cold complete medium, and then, the cells were washed with complete medium three times and used for further experiments.

### **Cell–cell contact cultures**

MSCs or AhR-activated MSCs were co-cultured with splenocytes using a cell–cell contact approach. Different ratios of MSCs or AhR-activated MSCs and splenocytes (0, 1:50, 1:10, and 1:5) were loaded to a 24-well, round-bottom plate. After the adherence of MSCs or AhR-activated MSCs to the plate wall, CFSE-labeled splenocytes at a concentration of 1 × 10^6^/well were added to the 24-well culture plate and concanavalin A (ConA) (eBioscience) was mixed at a concentration of 3 μg/mL to stimulate the proliferation of splenocytes. The CFSE-labeled splenocytes alone with no ConA were used as the negative control. These cells were cultured for 3 d, and the proliferation of cells was characterized by FACS.

### **Transwell culture**

A transwell culture system (0.4-μm Pore Polycarbonate Membrane Insert, 24-wells) (Corning) was used to prevent MSCs or AhR-activated MSCs from contacting splenocytes. MSCs or AhR-activated MSCs were loaded to the lower chamber, and splenocytes were added to the upper chamber at different ratios similar to those in the cell–cell contact culture system. ConA was also added to stimulate splenocyte proliferation. These cells were cultured for 3 d, and the proliferation was characterized by FACS.

### **RNA isolation and quantitative real-time PCR**

The total RNA from MSCs or AhR-activated MSCs was extracted using the TRIzol Reagent (Invitrogen, Waltham, MA). Reverse transcription of RNA to cDNA was conducted using the PrimeScript TM RT reagent kit (Takara, Dalian, China), and the polymerase chain reaction (PCR) was performed using the SYBR Premix Ex Taq TM II kit (Takara) to amplify the inducible nitric oxide synthase (iNOS)–encoding regions to determine the gene expression level, which was normalized to that of the endogenous reference gene, *GAPDH*. The forward and reverse primers of iNOS used were as follows: 5′-CCGAAGCAAACATCACATTCA-3′ and 5′-GGTCTAAAGGCTCCGGGCT-3′.

### **Western blot analysis**

Cells were resuspended in lysis buffer (Beyotime Institute of Biotechnology, Shanghai, China) for 30 min at 4°C. The lysate was centrifugated at 14,000 rpm for 15 min. The protein concentration of each sample was determined using the bicinchoninic acid protein assay (Beyotime Institute of Biotechnology). Equivalent amounts of protein were electrophoresed on an 8% gradient sodium dodecylsulfate-polyacrylamide gel and transferred to a polyvinylidenedifluoride membrane. The membranes were blocked with 5% skimmed milk at 25°C for 1 h and incubated with primary antibodies overnight at 4°C. The antibodies used were β-actin (Beyotime Institute of Biotechnology) and AhR and iNOS (Boster, Wuhan, China). The blots were incubated with a horseradish peroxidase–conjugated secondary antibody (Beyotime Institute of Biotechnology). The protein expression signals were detected with the enhanced chemiluminescence reagent (Millipore, Billerica, MA). ChemiDoc XRS+System was applied for protein relative quantification, and Image Lab 5.0 software was used for the analysis. β-actin was used as an internal reference protein.

### **Determination of NO concentration in the co-cultures**

The NO concentration was determined in the cell–cell contact and transwell cultures. NO is very active and quickly transformed into NO_3_- and NO_2_- in the medium, and NO_2_- is further transformed into NO_3_-. We measured NO_2_- production using the NO assay kit (Nanjing Jiancheng Bioengineering Institute, Nanjing, China), which is based on the principle of conversion of NO_3_- to NO_2_- with a nitrate reductase method, and the chromogenic depth was determined by multiscan spectrum. In order to investigate the effect of splenocytes on MSCs, MSCs were co-cultured with splenocytes in a 6-well plate. Protein of MSCs was extracted, and NO concentration was detected.

### **Determination of the effect of the NO donor S-nitroso-N-acetylpenicillamine, iNOS inhibitor N(G)-nitro L-arginine methyl ester, and NO scavenger 2-phenyl-4,4,5,5-tetramethylimidazoline-1-oxyl 3-oxide on splenocyte proliferation**

The CFSE-labeled splenocytes were activated with ConA in the absence of MSCs and in the presence of 100 μM S-nitroso-N-acetylpenicillamine (SNAP) (Sigma-Aldrich, St.Louis, MO) in a 24-well culture plate. With the standard condition (MSC:SPC = 1:10), splenocytes were activated with ConA in the presence of 1 mM N(G)-nitro L-arginine methyl ester (L-NAME) (Sigma-Aldrich) or 200 μM 2-phenyl-4,4,5,5-tetramethylimidazoline-1-oxyl 3-oxide (PTIO) (Sigma-Aldrich). Splenocyte proliferation was evaluated by FACS.

### **Statistical analyses**

Data are expressed as mean ± standard deviation, and all the statistical analyses were performed using SPSS Statistics 22.0 (IBM, Armonk, NY). Statistical significance was analyzed using the one-way analysis of variance and unpaired *t* test. The results with *P* < 0.05 were considered statistically significant.

## Results

### **Morphology and identification of MSCs**

**Fig. 1:**
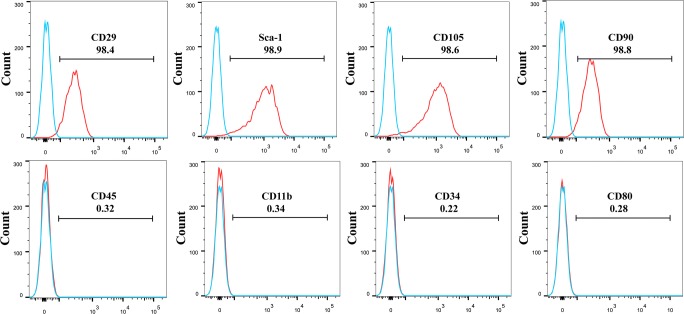
Identification of mesenchymal stem cells (MSCs) by fluorescence-activated cell sorting. MSCs were positive for CD29, Sca-1, CD105, and CD90 and negative for CD45, CD11b, CD34, and CD80

MSCs were plastic adherent and demonstrated fibroblast-like shape. The FACS analysis showed that MSCs were positive for CD29, Sca-1, CD105, and CD90 and negative for CD45, CD11b, CD34, and CD80 (Fig. 1).

### **Optimal mitomycin C concentration for the suppression of MSC proliferation**

**Fig. 2:**
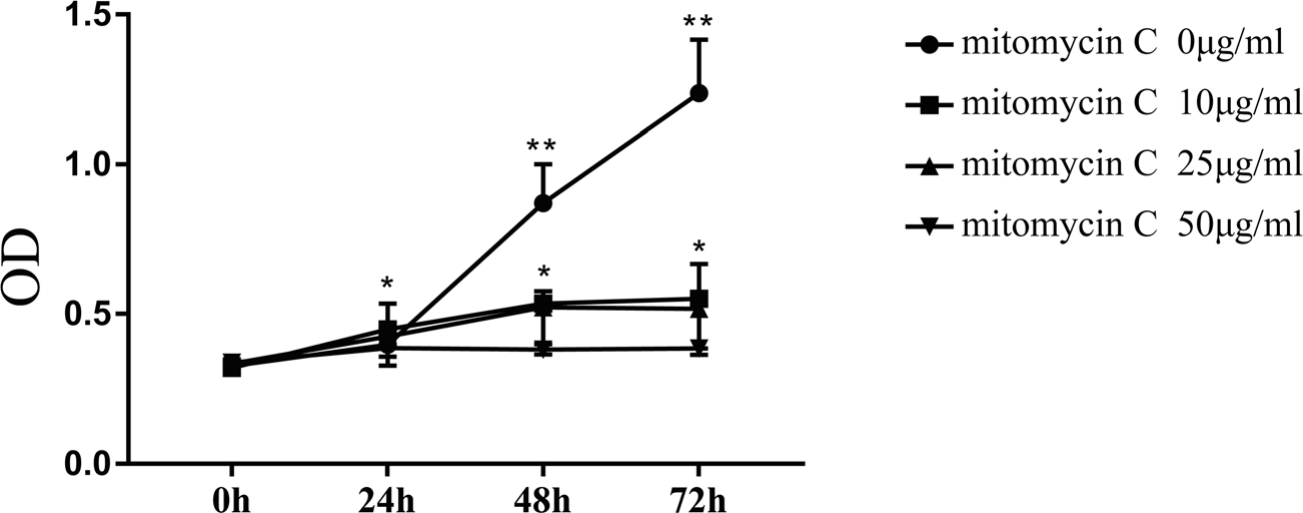
. Effects of different concentrations of mitomycin C on mesenchymal stem cell (MSC) proliferation. Treatment with mitomycin C at 50 μg/mL concentration in a water bath of 37°C for 30 min completely inhibited MSC proliferation. (Compared with the corresponding group of cells incubated for 0 h, *P < 0.05, **P < 0.01)

The results of the proliferation assay demonstrated that 50 μg/mL mitomycin C could completely inhibit MSC proliferation regardless of time (Fig. 2).

### **AhR activation enhanced the inhibitory effect of MSCs on splenocyte proliferation in the cell–cell contact and transwell cultures**

**Fig. 3:**
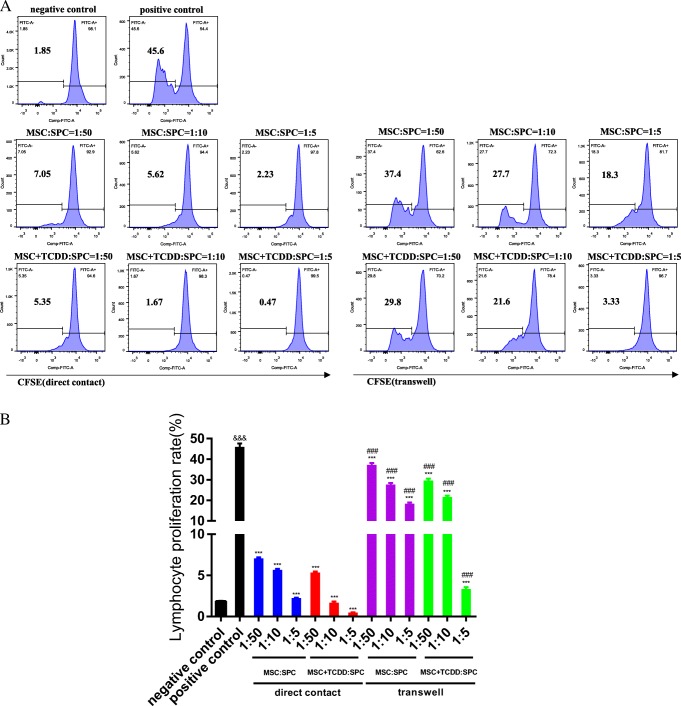
Inhibitory effect of mesenchymal stem cells (MSCs) and AhR-activated MSCs (MSC+TCDD) on the proliferation of splenocytes (SPC). MSCs or AhR-activated MSCs were co-cultured with splenocytes at different ratios in cell–cell contact and transwell system. The results were analyzed by fluorescence-activated cell sorting through the detcetion of CFSE. (*Ampersand* represents comparison with the negative control; *asterisk* represents comparison with the positive control; *number sign* represents comparison with the corresponding group of the same proportion in the cell–cell contact system, ***P < 0.001, ^###^*P <* 0.001)

We induced splenocyte proliferation using ConA, which has the ability to induce the mitogenic activity of splenocytes and synthesis of cellular products. As shown in Fig. 3, the inhibitory effect of MSCs or AhR-activated MSCs was enhanced with increase in the ratio of MSCs–splenocytes in the two co-culture systems, while AhR-activated MSCs augmented the suppressive function against splenocyte proliferation with the same ratio. The inhibitory effect of MSCs or AhR-activated MSCs on splenocyte proliferation in the cell–cell contact culture system was more distinct than that in the transwell culture system.

### **AhR activation upregulated the expression of inducible nitric oxide syntha**se

**Fig. 4:**
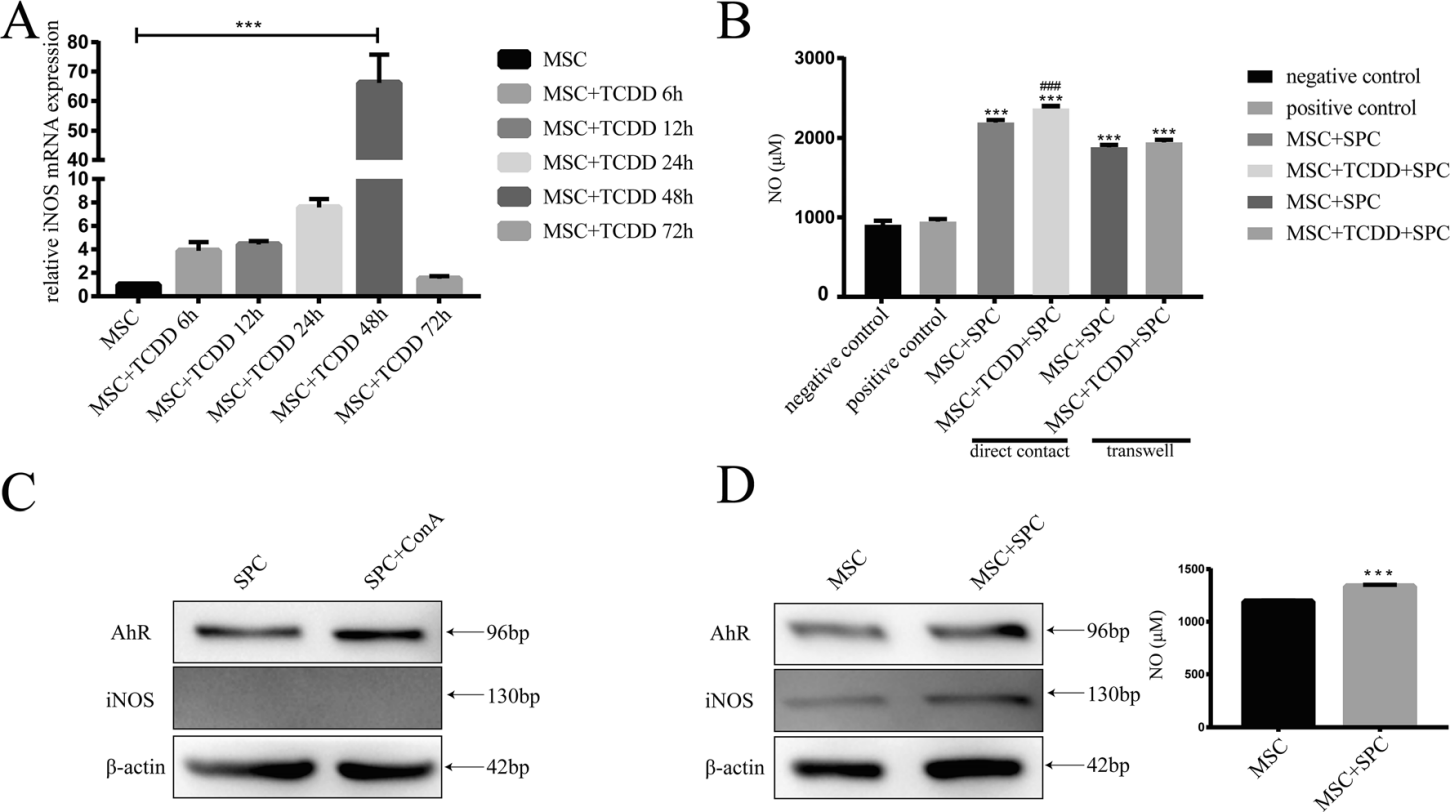
Expression of inducible nitric oxide (iNOS) upon AhR activation and detection of nitric oxide (NO). AhR activation upregulated iNOS (*A*) and enhanced NO production in the cell–cell contact and transwell co-culture systems (*B*). Splenocyte activation with ConA had no effect on the expression of AhR and iNOS (*C*). Splenocytes could stimulate the production of NO in MSCs and upregulate the iNOS while the AhR not (*D*)

A previous study demonstrated that mouse-derived MSCs suppressed splenocyte proliferation in a NO-dependent manner, which was regulated by iNOS (Sato et al. 2007). The experiment above has showed that AhR activation enhanced the suppressive function of MSCs against splenocyte proliferation. We performed a real-time PCR to evaluate the expression of iNOS with the activation of AhR in MSCs. The results demonstrated that the expression of iNOS peaked at 48 h and quickly dropped at 72 h (Fig. 4*A*).

### **AhR activation upregulated NO production**

As shown in Fig. 4*A*, AhR activation upregulated the expression of iNOS in MSCs, which is responsible for NO production. With the standard MSC:splenocyte ratio of 1:10, NO could be detected in both the cell–cell contact and transwell cultures, but the NO concentration in the former was relatively higher (Fig. 4*B*). Furthermore, AhR activation increased NO production in MSCs, which is in accordance with the inhibition of proliferation. Splenocytes treated with ConA proliferated significantly, and no increase in NO concentration was observed. Western blot analysis showed that there was no change of AhR and no expression of iNOS (Fig. 4*C*). In the co-culture system, splenocytes could stimulate the production of NO in MSCs (Fig. 4*D*). Western blot results showed that the iNOS increased in the co-cultured MSCs while the AhR did not.

### **Nitric oxide donor SNAP inhibited, whereas the iNOS inhibitor L-NAME and NO scavenger PTIO restored splenocyte proliferation**

**Fig. 5:**
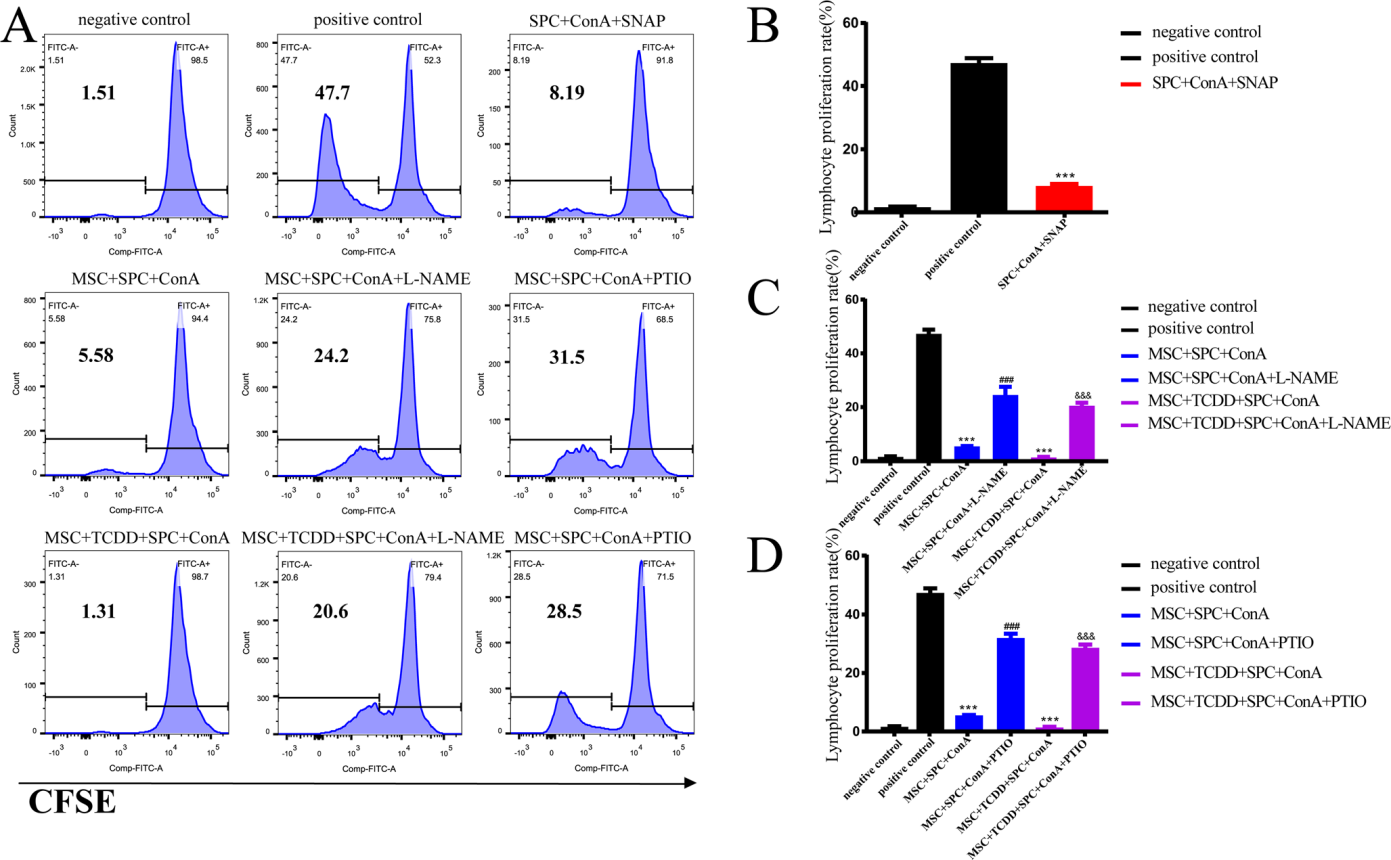
Effect of SNAP, L-NAME, and PTIO on the proliferation of splenocytes. The results were analyzed by fluorescence-activated cell sorting (*A*). The NO donor SNAP inhibited (*B*), whereas the iNOS inhibitor L-NAME (*C*) and NO scavenger PTIO (*D*) restored splenocyte proliferation (*asterisk* represents comparison with the positive control, *number sign* represents comparison with the MSC + SPC + ConA group, and *ampersand* represents comparison with the MSC + TCDD + SPC + ConA group. ***P < 0.001, ^###^*P <* 0.001, ^&&&^*P <* 0.001)

The results revealed that NO might be involved in the negative regulation of splenocyte proliferation. We used SNAP, which is widely used as a NO donor, to treat splenocytes activated by ConA for 72 h. SNAP significantly inhibited splenocyte proliferation (Fig. 5). The addition of L-NAME partially reversed the inhibitory effect of MSCs or AhR-activated MSCs. The results also demonstrated that PTIO treatment showed an effect similar to that of L-NAME.

## Discussion

Various physiological and pathological processes such as immune and inflammatory responses are affected by MSCs, which have been employed successfully to treat various immune-related disorders in animal models and clinical settings (Li et al. 2012). MSCs are a double-edged sword in the regulation of immune responses. The immunosuppressive capacity of MSCs is not always achieved. Several studies have concluded that IFN-γ synergized with another cytokine, either TNF-α, IL-1α, or IL-1β, is required to prime MSCs to exert the immunosuppressive effect (Ren et al. 2008). The addition of proinflammatory cytokines promotes MSCs to release potent chemoattractants, which will attract splenocytes to MSCs for immunosuppression. In the present study, we used IFN-γ and TNF-α to prime MSCs or AhR-activated MSCs in the experiments.

Several molecules have been reported to be involved in MSC-mediated immunosuppression (Shi et al. 2012). Murine MSCs utilize NO to inhibit immune responses. In the present study, the expression of iNOS upon the activation of AhR in MSCs and the results of NO detection assay indicated that AhR activation upregulated the expression of *iNOS* gene in MSCs, which promoted NO release, thus enhancing the inhibitory effect of MSCs on splenocyte proliferation. The effect was also associated with the ratio of MSCs or AhR-activated MSCs and splenocytes. The inhibition effect was enhanced when the ratio increased. MSCs or AhR-activated MSCs co-cultured with splenocytes at different ratios in the cell–cell contact culture showed a more intense inhibitory effect. NO is a kind of labile small molecule with extensive biological activity, which diminishes markedly over a distance of few-cell diameter (Porterfield et al. 2001). This might explain why a semi-permeable membrane might partly abrogate immunosuppression. iNOS can be induced by cytokines and other stimuli in many cell types, and it is calcium independent. iNOS is expressed in activated CD4+ T cell treated with CD3 and CD28 antibody (Jianjun et al. 2013). Other literature found that the induction of iNOS was detected in MSCs co-cultured with activated splenocytes treated by ConA but not in splenocytes alone (Sato et al. 2007). MSCs were the producer of NO in the setting of the experiment, which was in accordance with our results. Activation of the AhR leads to the degradation through the ubiquitination pathway. As showed in Fig. 4*D*, no activation of the AhR was observed after co-cultivation of MSCs with splenocytes, but iNOS and NO were upregulated. It was speculated that splenocytes promoted the expression of iNOS through other pathways, while MSCs further amplified their immunosuppressive effect through AhR–iNOS–NO pathway after treatment with TCDD.

As a NO donor, SNAP inhibited splenocyte proliferation, which further validated the role of NO in immunosuppression. With the standard ratio of fixed MSCs–splenocytes, the iNOS inhibitor L-NAME and NO scavenger PTIO partially reversed the inhibitory effect of MSCs or AhR-activated MSCs. The recovery was approximately 50% compared with that of the positive control. This suggests that other mediators, such as PGE2 and TSG-6, might be involved in the suppression effect of MSCs under stringent condition in this experiment. A number of molecules might be involved in a synergistic manner for MSCs to cause immunosuppression, and the identification of the interaction network might provide more references for clinical application.

Fibroblasts participate in airway remodeling in asthma. There was literature showing that AhR expression was higher in airway fibroblasts from asthmatic subjects as compared with healthy controls (Zhou et al. 2014). It also had been reported that distal lung fibroblasts are one source of synthesized alveolar NO. The data showed that fibroblasts from asthmatic patients revealed an increased iNOS expression compared with the control patients (Larsson-Callerfelt et al. 2015). Treatment with the selective iNOS inhibitor significantly increased synthesis of the proteoglycan versican. We speculate that there is an AhR–iNOS–NO activity in fibroblasts from asthmatic patients, which could withstand the pathological airway remodeling.

Some studies have reported a dysfunction of MSCs in animal models, which might be associated with the clinical setting and microenvironment, which might dampen the immunosuppression property of MSCs (Cheng et al. 2017; Fijany et al. 2019). Previously, we found that downregulation of AhR in MSCs isolated from mice with neutrophilic asthma and AhR activation enhanced the function of MSCs to alleviate neutrophilic airway inflammation by inhibiting Th17 polarization. Modification of MSCs might provide an approach to augment their effect on immune responses.

Our study showed that AhR activation enhanced the function of MSCs to inhibit splenocyte proliferation in vitro in a NO-dependent manner; however, further studies should be conducted to elucidate the underlying mechanisms. As described above, human MSCs exert an immunosuppressive effect through a different molecule, IDO. Enhancement of AhR activation resulted in the upregulation of IDO enzyme, which is involved in the generation of kynurenic acid and xanthurenic acid, both of which act as AhR ligands (Heath-Pagliuso et al. 1998). These findings suggest that AhR activation might lead to a positive feedback loop that prolongs or amplifies AhR signaling in the local environment (Quintana and Sherr 2013). Recently, it has been reported that, in a mouse model of lipopolysaccharide-induced acute lung injury with increased number of total cells and neutrophils in the bronchoalveolar lavage fluid, pretreatment of human MSCs with kynurenic acid enhanced the therapeutic effect of MSCs on ALI and controlled their anti-inflammatory therapeutic effects mediated by TSG-6 (Wang et al. 2018). The study demonstrated a potent application of AhR in modifying human MSCs in clinical settings.

In the present study, we found that AhR activation upregulated iNOS expression and NO production in MSCs, thereby enhancing their immunosuppressive ability. A previous study reported that phosphorylation of signal transducers and activators of transcription 5 (STAT5) might be associated with NO and might be involved in the proliferation of splenocytes (Sato et al. 2007). However, further studies are needed to determine the underlying mechanism of the involvement of the NO-STAT5 pathway and cell cycle–related proteins in the proliferation of splenocytes.

## Conclusion

Our study demonstrated that TCDD-induced AhR activation enhanced the suppressive function of MSCs against splenocyte proliferation, providing valuable information to develop a new treatment option for immune-related diseases. Approaches to activate the AhR pathway could potentially increase the efficiency of MSC-based therapies in the future.

## References

[CR1] Cella M, Colonna M (2015). Aryl hydrocarbon receptor: linking environment to immunity. Semin Immunol.

[CR2] Cheng Y, Gu W, Zhang G, Li X, Guo X (2017). Activation of Notch1 signaling alleviates dysfunction of bone marrow-derived mesenchymal stem cells induced by cigarette smoke extract. Int J Chron Obstruct Pulmon Dis.

[CR3] de Araujo Farias V, Carrillo-Galvez AB, Martin F, Anderson P (2018). TGF-beta and mesenchymal stromal cells in regenerative medicine, autoimmunity and cancer. Cytokine Growth Factor Rev.

[CR4] Fijany A, Sayadi LR, Khoshab N, Banyard DA, Shaterian A, Alexander M, Lakey JRT, Paydar KZ, Evans GRD, Widgerow AD (2019). Mesenchymal stem cell dysfunction in diabetes. Mol Biol Rep.

[CR5] Heath-Pagliuso S, Rogers WJ, Tullis K, Seidel SD, Cenijn PH, Brouwer A, Denison MS (1998). Activation of the Ah receptor by tryptophan and tryptophan metabolites. Biochemistry.

[CR6] Jianjun Y, Zhang R, Lu G, Shen Y, Peng L, Zhu C, Cui M, Wang W, Arnaboldi P, Tang M, Gupta M, Qi CF, Jayaraman P, Zhu H, Jiang B (2013). T cell-derived inducible nitric oxide synthase switches off Th17 cell differentiation. J Exp Med.

[CR7] Kim JS, Cha SH, Kim WS, Han SJ, Cha SB, Kim HM, Kwon KW, Kim SJ, Choi HH, Lee J, Cho SN, Koh WJ, Park YM, Shin SJ (2016). A novel therapeutic approach using mesenchymal stem cells to protect against Mycobacterium abscessus. Stem Cells.

[CR8] Larsson-Callerfelt AK, Weitoft M, Nihlberg K, Bjermer L, Westergren-Thorsson G, Tufvesson E (2015). iNOS affects matrix production in distal lung fibroblasts from patients with mild asthma. Pulm Pharmacol Ther.

[CR9] Li W, Ren G, Huang Y, Su J, Han Y, Li J, Chen X, Cao K, Chen Q, Shou P, Zhang L, Yuan ZR, Roberts AI, Shi S, Le AD (2012). Mesenchymal stem cells: a double-edged sword in regulating immune responses. Cell Death Differ.

[CR10] Lin CS, Lin G, Lue TF (2012). Allogeneic and xenogeneic transplantation of adipose-derived stem cells in immunocompetent recipients without immunosuppressants. Stem Cells Dev.

[CR11] Meisel R, Zibert A, Laryea M, Gobel U, Daubener W, Dilloo D (2004). Human bone marrow stromal cells inhibit allogeneic T-cell responses by indoleamine 2,3-dioxygenase-mediated tryptophan degradation. Blood.

[CR12] Milosavljevic N, Gazdic M, Simovic Markovic B, Arsenijevic A, Nurkovic J, Dolicanin Z, Jovicic N, Jeftic I, Djonov V, Arsenijevic N, Lukic ML, Volarevic V (2018). Mesenchymal stem cells attenuate liver fibrosis by suppressing Th17 cells - an experimental study. Transpl Int.

[CR13] Najar M, Raicevic G, Fayyad-Kazan H, Bron D, Toungouz M, Lagneaux L (2016). Mesenchymal stromal cells and immunomodulation: a gathering of regulatory immune cells. Cytotherapy.

[CR14] Porterfield DM, Laskin JD, Jung SK, Malchow RP, Billack B, Smith PJ, Heck DE (2001). Proteins and lipids define the diffusional field of nitric oxide. Am J Phys Lung Cell Mol Phys.

[CR15] Quintana FJ, Sherr DH (2013). Aryl hydrocarbon receptor control of adaptive immunity. Pharmacol Rev.

[CR16] Ren G, Su J, Zhang L, Zhao X, Ling W, L'Huillie A, Zhang J, Lu Y, Roberts AI, Ji W, Zhang H, Rabson AB, Shi Y (2009). Species variation in the mechanisms of mesenchymal stem cell-mediated immunosuppression. Stem Cells.

[CR17] Ren G, Zhang L, Zhao X, Xu G, Zhang Y, Roberts AI, Zhao RC, Shi Y (2008). Mesenchymal stem cell-mediated immunosuppression occurs via concerted action of chemokines and nitric oxide. Cell Stem Cell.

[CR18] Sato K, Ozaki K, Oh I, Meguro A, Hatanaka K, Nagai T, Muroi K, Ozawa K (2007). Nitric oxide plays a critical role in suppression of T-cell proliferation by mesenchymal stem cells. Blood.

[CR19] Schmidt JV, Bradfield CA (1996). Ah receptor signaling pathways. Annu Rev Cell Dev Biol.

[CR20] Shi Y, Su J, Roberts AI, Shou P, Rabson AB, Ren G (2012). How mesenchymal stem cells interact with tissue immune responses. Trends Immunol.

[CR21] Su J, Chen X, Huang Y, Li W, Li J, Cao K, Cao G, Zhang L, Li F, Roberts AI, Kang H, Yu P, Ren G, Ji W, Wang Y, Shi Y (2014). Phylogenetic distinction of iNOS and IDO function in mesenchymal stem cell-mediated immunosuppression in mammalian species. Cell Death Differ.

[CR22] Uder C, Bruckner S, Winkler S, Tautenhahn HM, Christ B (2018). Mammalian MSC from selected species: features and applications. Cytometry A.

[CR23] Wang G, Cao K, Liu K, Xue Y, Roberts AI, Li F, Han Y, Rabson AB, Wang Y, Shi Y (2018). Kynurenic acid, an IDO metabolite, controls TSG-6-mediated immunosuppression of human mesenchymal stem cells. Cell Death Differ.

[CR24] Zhou Y, Mirza S, Xu T, Tripathi P, Plunkett B, Myers A, Gao P (2014). Aryl hydrocarbon receptor (AhR) modulates cockroach allergen-induced immune responses through active TGFbeta1 release. Mediat Inflamm.

